# Anti-Cancer and Anti-Oxidant Bioactive Metabolites from *Aspergillus fumigatus* WA7S6 Isolated from Marine Sources: In Vitro and In Silico Studies

**DOI:** 10.3390/microorganisms12010127

**Published:** 2024-01-08

**Authors:** Mervat G. Hassan, Waleed A. Elmezain, Dina M. Baraka, Sabah A. AboElmaaty, Ahmed Elhassanein, Riyad Mohammed Ibrahim, Ahmed A. Hamed

**Affiliations:** 1 Botany and Microbiology Department, Faculty of Science, Benha University, Benha 33516, Egypt; mervat.hassan@fsc.bu.edu.eg (M.G.H.); waliedahmed89@yahoo.com (W.A.E.); dina.barakah@fsc.bu.edu.eg (D.M.B.); sabah.alsayed@fsc.bu.edu.eg (S.A.A.); 2 Department of Mathematics, College of Science, University of Bisha, P.O. Box 551, Bisha 61922, Saudi Arabia; rmebrahim@ub.edu.sa; 3 Microbial Chemistry Department, National Research Centre, El-Buhouth St. 33, Cairo 12622, Egypt

**Keywords:** *Aspergillus fumigatus*, bioactive metabolites, in silico study

## Abstract

Cancer is a huge global disease burden. Every year, tens of millions of people worldwide are diagnosed with cancer, and more than half of them die as a result of it. The great biodiversity of the marine environment has increasingly piqued the interest of experts, especially in the field of drug discovery. The marine fungus *Aspergillus fumigatus* WA7S6 has been selected among a group of fungi isolated from marine sponges as it exhibits a pronounced antimicrobial activity toward a group of pathogenic microbes. The fungus has been identified genetically by amplification and analysis of its 18srRNA gene. The fungus crude extract has been obtained by cultivation of the fungus on rice media. The crude extract was tested for antibacterial activity against a variety of pathogenic microorganisms. The results demonstrated a pronounced antimicrobial action against *P. aeruginosa*, *S. aureus*, *A. niger*, and *Candida albicans*. Furthermore, we tested the antioxidant potential of the Aspergillus fumigatus WA7S6 crude extract using three different methods: ATBS, DPPH, and lipid peroxidation assays. Results showed that the crude extract WA7S6 had an IC50 value of 21.35 µg/mL. The anticancer potential of the crude extract was also evaluated against cancer cell lines such as Hela, MCF, and WI-38. The chemical profiling of the fungus extract was identified via GC-mass and in silico molecular docking of the identified compounds on heme oxygenase, as a stress protein included in cellular protection, antioxidant, and anti-inflammatory activities, suggesting that some compounds, such as 9-Tetradecynoic acid, 11-Hexadecynoic acid, methyl ester, and dehydromevalonic lactone, could be relevant for antioxidant purposes.

## 1. Introduction

Marine natural products are key sources of biologically active molecules that have been shown to modulate a variety of biological functions, including antioxidant, antimicrobial, and anticancer properties [1]. In their pursuit of novel cancer treatments, scientists and medical researchers have looked to nature for prospective medicines. The interesting realm of endophytic fungus is one of the potential areas of inquiry [2]. Endophytes are symbiotic microorganisms that live in plant tissues, frequently without being harmful to their hosts. Surprisingly, these fungi have demonstrated the capability to synthesize a diverse spectrum of bioactive molecules with several biological effects [3]. 

Marine fungi, which are generally overlooked in favor of their terrestrial counterparts, have emerged as a promising source of bioactive chemicals, some of which have extraordinary antioxidant and anticancer capabilities [4]. *Aspergillus versicolor*, a marine-derived Aspergillus species, has been identified as being able to generate bioactive chemicals with significant antioxidant potential. Aspergiolide A has shown substantial antioxidant activity with an IC_50_ value of 4.57 M, making it a tempting option for fighting oxidative stress [5]. *Aspergillus versicolor* is also known for its ability to produce compounds with anticancer activities. A well-known example is versicolactone B, which has displayed a potent cytotoxic activity toward cancer cell lines, with an IC_50_ value of 2.47 µM, indicating its possible application as an anticancer agent [6].

Another example is the marine-derived *Aspergillus sydowii*, which produces bioactive compounds with significant antioxidant capabilities. For instance, the compound sydonic acid, derived from *Aspergillus sydowii*, has demonstrated potent antioxidant activity, with an IC50 value of 9.52 µM [7]. Moreover, this fungus has been reported for its ability to produce sydowic acid, which displayed a promising cytotoxic effect on cancer cells, with an IC_50_ value of 5.83 µM, highlighting the importance of marine-derived *Aspergillus* sp. in anticancer drug discovery [8]. Fumitremorgin C, a molecule produced from the marine fungus *Aspergillus fumigatus*, has shown significant antioxidant effects. It effectively scavenges free radicals, protecting cells from oxidative damage. Furthermore, there is rising hope about FTC’s potential as an anticancer agent. It displays cytotoxic effects on a variety of cancer cell lines, showing its potential as a promising option for anticancer medication development [9].

The main goal of this study was to isolate endophytic fungi living within marine sponges and seagrass. Following that, we intended to thoroughly examine these fungi’s ability to produce bioactive molecules with the ability to treat cancer and exhibit significant antioxidant qualities. This multimodal investigation aims to shed insight into the recovered chemicals’ cytotoxic and antioxidant properties, suggesting intriguing pathways for future biological and pharmacological uses.

## 2. Materials and Methods

### 2.1. Sampling

Marine sponge samples were collected from Hurghada, a coastal city renowned for its diverse marine ecosystems. The collection was conducted using SCUBA diving-trained divers, while underwater, and they meticulously identified and gathered sponges from different substrates, including seagrass and sponges, at each location. After collection, the samples were assigned unique codes, photographed, and then deposited at the Microbial Chemistry Department in Egypt [10,11].

### 2.2. Isolation of Associated Fungi from Sponge Samples

Isolation of associated fungi was started by surface sterilization of the collected marine samples; the sponges were initially cleaned with tap water before being surface-sterilized with a series of treatments that included 70% ethanol, sterile distilled water, 2% sodium hypochlorite, and repeated distilled water rinses. The surface-sterilized sponge was then cut into small pieces and put on a potato dextrose agar (PDA) medium enriched with nalidixic acid and chloramphenicol to inhibit bacterial growth. The plates were then incubated at 28 °C until the appearance of fungal colonies. Individual fungal colonies were chosen, sub-cultured several times to assure purity, and stored in glycerol stocks at −20 °C at the Microbial Chemistry Dept. National Research Centre [1,12,13]. 

### 2.3. Genetic Identification of Selected Fungi

The fungal isolate’s 18S rDNA sequence was analyzed to confirm its identity. The fungal DNA extraction was performed after cultivation of the fungus in a 250 mL Erlenmeyer flask containing 50 mL of potato dextrose broth medium at 28 °C for 3 days. After incubation, the mycelia were removed and the DNA was extracted by the Qiagen DNeasy Mini Kit, USA [14]. The 18SrRNA gene was amplified using two global primers, NS3 (5′-GCAAGTCTGGTGCCAGCAGCC amplification 3′) and NS4 (5′-CTTCCGTCAATTCCTTTAAG-3′) [15]. The PCR profile was as follows: 5 min denaturation step at 94 °C, followed by 35 cycles of 94 °C for 30 s, 55 °C for 30 s, 72 °C for 90 s, and a 5 min extended step at 72 °C. Sequencing of the PCR product was conducted at the SolGent Company in South Korea. The obtained sequence was analyzed by BLAST to determine the homogeneity degree with all deposited sequences available in the database of the NCBI (National Centre Biotechnology Information). The phylogenetic tree was built by the MEGA7 software [16,17].

### 2.4. Fungal Cultivation and Large-Scale Production 

The isolates of the fungi were grown on rice media (100 g rice in 100 mL synthetic sea water, salinity reduced to 50%). Incubation was carried out at 28 °C for 15 days. After incubation, culture extraction was carried out by ethyl acetate (EtOAc), and then the EtOAc extract was vacuum-dried to obtain the crude extracts [18].

### 2.5. Gas Chromatography-Mass Spectrometry (GC-MS)

To identify the chemical composition of the fungal extract, Varian gas chromatography/mass spectrometry (Perkin Elmer Auto XL GC) with flame ionization detection was used for analysis. An EQUITY-5 column, H2 carrier gas, and precise temperature programming were used in the analysis. The mass range was *m*/*z* 39–400 amu, and the ionization voltage was 70 eV. Retention periods in comparison to real samples and matching spectral peaks from published data were used for compound identification.

### 2.6. Antimicrobial Screening

The antimicrobial activity of the fungal crude extracts was evaluated against various microorganisms, including penicillin-resistant *E. coli*, *C. albicans*, *A. niger*, and *S. aureus*, using a 96-well polystyrene microplate assay [1,19]. Ciprofloxacin (10 µg/mL) and Nystatin (5 µg/mL) were used as positive reference antibiotics. All test pathogens were obtained from the Culture Collection Center (Microbial Chemistry Department and National Research Centre, NRC), Egypt.

### 2.7. Anticancer Screening

#### 2.7.1. Cell lines and Culture Media

Tumor and normal cell lines were obtained from Sigma-Aldrich in the United States. The cells were cultured according to the supplier’s suggestions in high glucose RPMI 1640 media with, penicillin (100 U/mL), streptomycin (100 μg/mL), and 10% FBS (Thermo Fisher Scientific Inc., Waltham, MA, USA) and then the incubation was carried out at 37 °C in a humid atmosphere (5% CO_2_).

#### 2.7.2. Cytotoxicity Assay

The cytotoxicity of the fungal crude extract was assessed using the 3-(4,5-Dimethylthiazol-2yl)-2,5-diphenyl tetrazolium bromide (MTT) assay, which was significantly modified from Van Loosdrecht et al. (1994) by Sigma-Aldrich, St. Louis, MO, USA. Propylene glycol (Sigma-Aldrich, USA) was employed to dissolve the AlNPs. The stocks were diluted in the culture medium to the appropriate concentration before use, with the final concentration of propylene glycol in each well at 0.1% (*v*/*v*). The cells in the control group were treated only with the vehicle. A 96-well tissue culture plate was seeded with 1 × 105 cells/mL (100 μL/well) and incubated at 37 °C for 24 h to form a complete monolayer. After the formation of a confluent cell monolayer, the growth medium was aspirated, and the monolayer was washed twice with washing solution. ALNPs were then diluted in RPMI medium supplemented with 2% maintenance serum medium. Subsequently, 0.1 mL of each dilution was added to various wells, with three control wells receiving only maintenance media. After incubation, the cells were examined for any signs of toxicity such as cell shrinkage, rounding, and complete or partial loss of the monolayer. An MTT solution (5 mg/mL in PBS) was prepared, and 20 µL of the solution was added to each well. The plate was placed on a shaker (150 rpm/5 min) to mix the MTT evenly in the medium. Incubation at 37 °C with 5% carbon dioxide was carried out for 1 to 5 h to allow MTT metabolism. For the dissolution of formazan (the metabolic product of MTT) crystals, formazan was thoroughly mixed in 200 µL of DMSO after removing the medium. An enzyme-linked immunosorbent assay (ELISA) plate reader was used to measure the absorbance at 570 nm to cellular density. The inhibitory concentration at half-maximal (IC50) was calculated using GraphPad Prism 8.2.4. Cell morphology was observed using a digital camera connected to a Nikon microscope. The experiments were repeated three times.

### 2.8. Antioxidant Activities

#### 2.8.1. DPPH Radical Scavenging Assay

The ability of the extracts to scavenge free radicals was assessed using the DPPH radical scavenging assay [20]. The extracts’ capacity to donate hydrogen atoms was determined by measuring the decolorization of a methanol solution of 2,2-diphenyl-1-picrylhydrazyl (DPPH). In the presence of antioxidants, DPPH changes from a violet/purple color in a methanol solution to yellow. A 0.1 mM DPPH methanol solution was prepared, and 2.4 mL of this solution was mixed with 1.6 mL of extract in methanol at various concentrations (100–1000 µg/mL). The reaction mixture was thoroughly vortexed and left in the dark at room temperature for 30 min. A spectrophotometer was then used to measure the absorbance at 517 nm. Vitamin C was used as a reference. The percentage of DPPH radical scavenging activity was calculated using the following equation:

% DPPH radical scavenging activity = [(A_0_ − A1)/A_0_] × 100

where A_0_ is the control absorbance and A1 is the extractives/standard absorbance. The IC_50_ was calculated by plotting the inhibition percentage against concentration. The experiment was performed in triplicate at every concentration.

#### 2.8.2. ABTS^•+^ Scavenging Assay

ABTS was used to assess the capacity for free radical scavenging (2,2′-Azino-bis-3-ethylbenzothiazoline-6-sulfonic acid) cation radical scavenging technique. Initially, a reaction between a 2.45 mM potassium persulfate solution and 2 mM ABTS in pure water was allowed to occur over 12 h at room temperature, generating ABTS radicals. The ABTS cation exhibits a prominent absorption peak at 734 nm and has a dark blue-green color. To achieve an absorption of 0.9 ± 0.1 at 734 nm, a phosphate buffer with a concentration of 0.1 mM and a pH of 7.4 was added to the ABTS cation solution. Three milliliters of extract solution were separately mixed with one milliliter of ABTS^•+^ solution at concentrations ranging from 10 to 30 mg/mL, along with standard compounds and methanol. After thorough vortexing, the samples were kept in a dark room for 30 min. Following this incubation, the absorbance at 734 nm was measured. A decrease in sample absorption indicates active ABTS^•+^ radical scavenging.

#### 2.8.3. Lipid Peroxidation in Ammonium Thiocyanate Medium

The lipid peroxidation inhibition assay was conducted following the method described by Haenen and Bast [21]. Thiobarbituric acid (TBA)-reactive species were responsible for the lipid peroxidation (LPO) activity. To induce LPO, 0.005 mL of FeSO_4_ (0.07 M) and approximately 1 mL of distilled water were added to the mixture, which was then incubated for 30 min. Afterward, the mixture was heated to 95 °C for 1 h. Subsequently, 1.5 mL of 0.8% (*w*/*v*) TBA, 1.5 mL of 20% acetic acid, 0.5 mL of 20% trichloroacetic acid (TCA), and 1.1% SDS were added. Another set of samples was treated similarly but without TBA. After cooling, each tube was filled with 5.0 mL of butanol and centrifuged for 10 min at 5000 rpm. The percentage of lipid peroxidation inhibition in the samples was calculated using the following formula:

% lipid peroxidation inhibition = [(A_0_ − A1)/A_0_] × 100

### 2.9. Molecular Docking

Using Molecular Operating Environment (MOE) software (MOE_2015), a molecular docking study was conducted on heme oxygenase for the identified chemicals from the ethyl acetate extract of Aspergillus. Heme oxygenase’s X-ray crystal structure (PDB code: 1N3U) was obtained via the Protein Data Bank website (www.pdb.org, accessed on 18 November 2023). The anticipated binding interactions and energy for each of the compounds were ascertained and compared.

### 2.10. Statistical Analysis

The SPSS V-20 software was used to compute and compare the means and SDs (standard deviations) of all experiments, which were all run in triplicate. One-way ANOVA was used to determine the differences’ significance at *p* ≤ 0.05. The IC_50_ values were obtained from a sigmoid-type nonlinear regression that was processed via the GraphPad 8.2.4^®^ program.

## 3. Results

### 3.1. Sample Collection and Isolation

The collection of marine samples was carried out in January 2022 from Hurghada City, Egypt. The samples were transferred carefully to the laboratory in a special sterilized container. The samples were coded, photographed, and kept in the fridge, until further processing. Table 1 represent the datasheet for the collected marine samples including location, depth, and codes. The undersea environment of the Red Sea is highly diverse, with 2100 fish species and over 300 coral species, 10% of which are exclusive to the area. Sponge diversity and sea grass are two of this ecosystem’s unsung heroes, acting as important sources of microorganisms, particularly fungi, in this active aquatic environment [22].

### 3.2. Isolation of Associated Fungi and Small-Scale Fermentation

Isolation of associated fungi from collected marine samples was performed after surface sterilization of the marine samples. Visual examination of these isolated fungal colonies included analyzing their morphological characteristics such as colony color, texture, and growth patterns, which led to the isolation of 13 isolates. The isolated fungi were classified into various taxa and groups based on their morphological features. The observed variations in colony morphology provided initial insights into the diversity of sponge-associated fungi (Supplementary Material S1). These morphologically differentiated fungi present a promising resource for further investigation and characterization, as illustrated in Table 2. The isolated fungal strains were cultivated on the rice medium to obtain the small crude extracts. The Fungal spore suspensions were inoculated and incubated for 15 days in 250 mL flasks containing 25 g solid rice media. After incubation, the cultures were extracted using ethyl acetate. After extraction, the ethyl extract was filtered using filter paper and completely evaporated to obtain the small-scale extracts for all isolated fungi as illustrated in Table 2 [23].

### 3.3. Antimicrobial Screening

The inhibitory activity of the fungal small-scale toward pathogenic bacteria, including gram-positive (*Staphylococcus aureus*), gram-negative (*P. aeruginosa*), and fungal (*C. albicans* and *A. niger*) species, was screened. The obtained results showed that only the WA7S6 extract exhibited a pronounced inhibition activity ratio. According to the obtained results, the inhibition ratio for *S. aureus* growth was 75.55 ± 0.21, the inhibition ratio for *P. aeruginosa, C. albicans,* and *A. niger* was 84.31 ± 0.35, 45.25 ± 1.26, and 65.42 ± 0.24, respectively. Additionally, the WA7S6 extract displayed no inhibition effects against *Salmonella typhi*. The obtained results showed that marine fungi are a potential source of antimicrobial agents with a variety of inhibitory activities. Additional investigation into the extract’s active ingredients may shed light on its modes of action and pave the way for the creation of novel therapeutic medicines that are effective against a variety of infections (Table 3).

### 3.4. Genetic Identification of the Most Potent Isolate 

**Figure 1:**
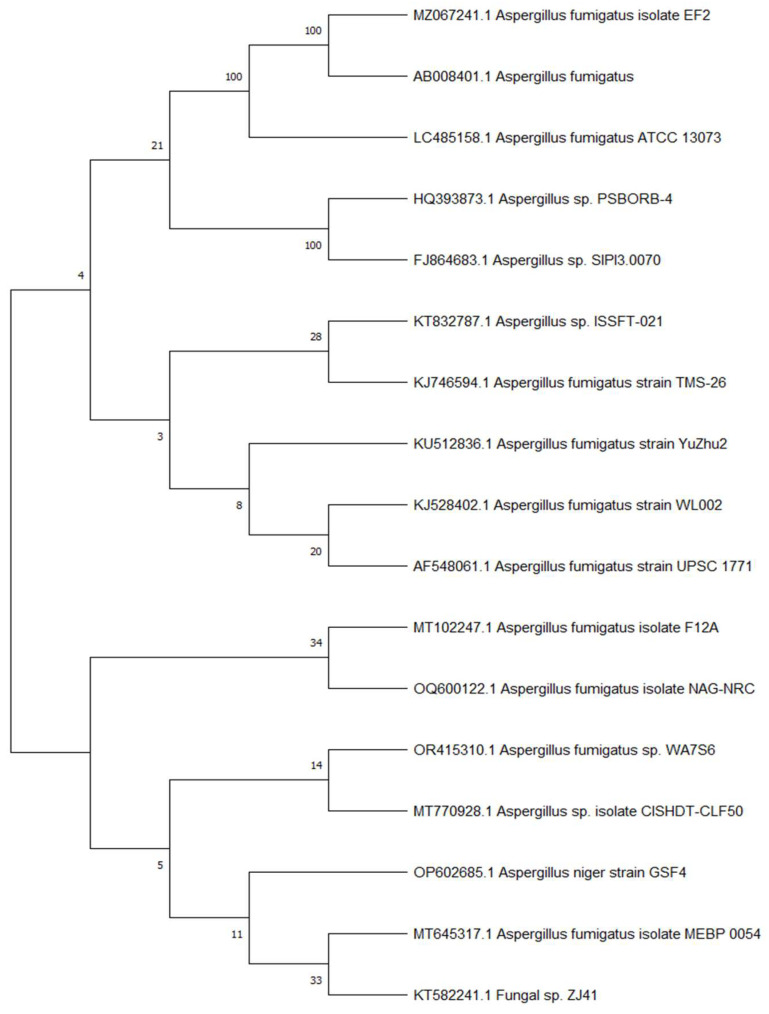
Phylogenetic tree of *Aspergillus fumigatus* WA7S6.

The most potent isolate, (WA7S6), was selected based on antimicrobial screening and identified genetically by amplification of its 18S rRNA gene. The identified sequence of 18S rRNA was then analyzed and aligned via BLAST with deposited known sequences found in GenBank to determine the similarity scores and statistically significant disparities among the matches. The obtained results showed a high degree of similarity, with the *Aspergillus fumigatus* exhibiting a homology of 100%. Construction of the phylogenetic tree was performed using the neighbor-joining approach [24] (Figure 1). The reliability of the tree’s topology was evaluated through a bootstrap test involving 500 iterations, indicating the proportion of times the connected taxa clustered together alongside the branches. The tree was constructed to scale, with lengths of branches reflecting developmental distances estimated through the method of Maximum Composite Likelihood [25]. The software MEGA 7 was employed for the execution of evolutionary analysis. Combining the obtained analysis data, the WA7S6 isolate was identified as *Aspergillus fumigatus* WA7S6. Therefore, the strain has been officially deposited at GenBank, under accession number OR415310.1.

### 3.5. Gas Chromatography of the Aspergillus fumigatus Crude Extract

Gas chromatography–mass spectrometry (GC-MS) analysis of the *Aspergillus fumigatus* crude extract showed the presence of numerous bioactive metabolites at different retention times including ethyl (L)-(-)-lactate, 2,3-Butanediol, 9,12-Octadecadien-1-ol, 13-Docosenamide, Bis(2-ethylhexyl) phthalate, Dimethylethylene glycol, and here major compounds detected dehydromevalonic lactone (C1), 11-Hexadecynoic acid, methyl ester (C2), and 9-Tetradecynoic acid (C3) (Supplementary Material S2).

The presence of dehydromevalonic lactone in the GC-MS profile suggests an intriguing link to the mevalonate pathway, a critical metabolic process responsible for the formation of many isoprenoid chemicals, while the mevalonate pathway is also involved in both primary and secondary metabolism [26]. The identification of 11-Hexadecynoic acid, methyl ester in the GC-MS profile showed the lipid diversity of *Aspergillus fumigatus*. This compound is a fatty acid with probable bioactive properties, indicating that lipid metabolism is involved in fungal cellular processes. Methyl ester may shed light on the intricate interactions between fungal lipids and their environment by revealing the role of 11-Hexadecynoic acid, potentially changing membrane integrity, signaling, and virulence [27].

The detection of 9-Tetradecynoic acid also indicates the lipid repertoire of *Aspergillus fumigatus*. Alkynes are versatile components that can be important in a variety of chemical reactions, and they may be important in the fungus’s biological processes. This improved understanding of *Aspergillus fumigatus*’ metabolic capabilities adds to our understanding of fungal biology and may pave the way for the development of new therapeutic or industrial applications.


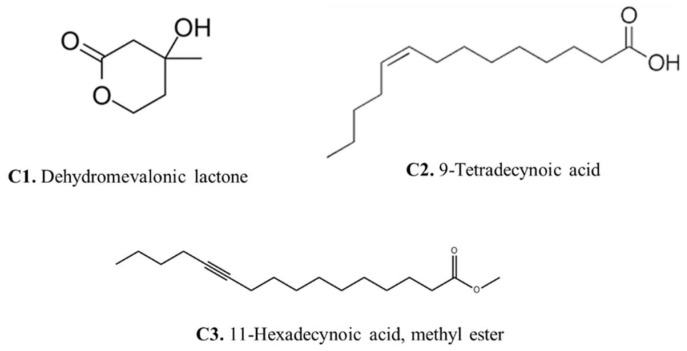


### 3.6. Biological Evaluation of Aspergillus fumigatus sp. AW7S6

#### 3.6.1. Antioxidant Activity

**Figure 2:**
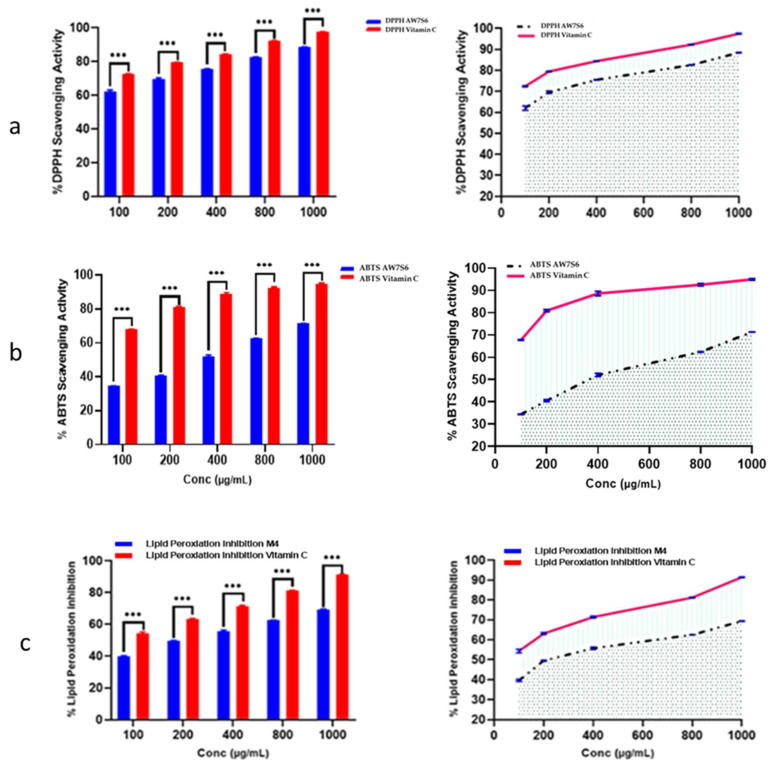
Antioxidant activity of AW7S6 using (**a**) DPPH, (**b**) ABTS, and (**c**) Lipid peroxidation •••: strong significance. Data were obtainable as mean ± SD. Wilcoxon–Mann–Whitney U test was used to analyze data. One-way ANOVA-*t*-test was used to make a comparison between the two groups.

Oxidation is a chemical reaction in which electrons or hydrogen are transferred from a substance to an oxidizing agent, resulting in the formation of free radicals. When these radicals enter a cell, they can set off a chain reaction that causes cellular damage or death. The antioxidant capacity of *Aspergillus fumigatus* WA7S6 crude extract was rigorously estimated using three well-established approaches: ABTS radical scavenging assay, DPPH radical scavenging assay, and the lipid peroxidation assay. The crude extract of WA7S6 had an IC50 value of 21.35 µg/mL in this study, indicating the concentration at which it effectively reduced oxidative stress by 50% across all three tested assays. This numerical value not only demonstrates WA7S6’s significant antioxidant potential but also allows for a meaningful comparison with established standards. Vitamin C, a well-known antioxidant, was used as a reference standard to provide a baseline for this assessment. Under the same experimental conditions, the IC50 value for Vitamin C was determined to be 9.69 µg/mL. This direct comparison highlights WA7S6’s antioxidant activity in comparison to a well-known antioxidant compound like Vitamin C (Figure 2).

#### 3.6.2. Anticancer Activity 

**Figure 3:**
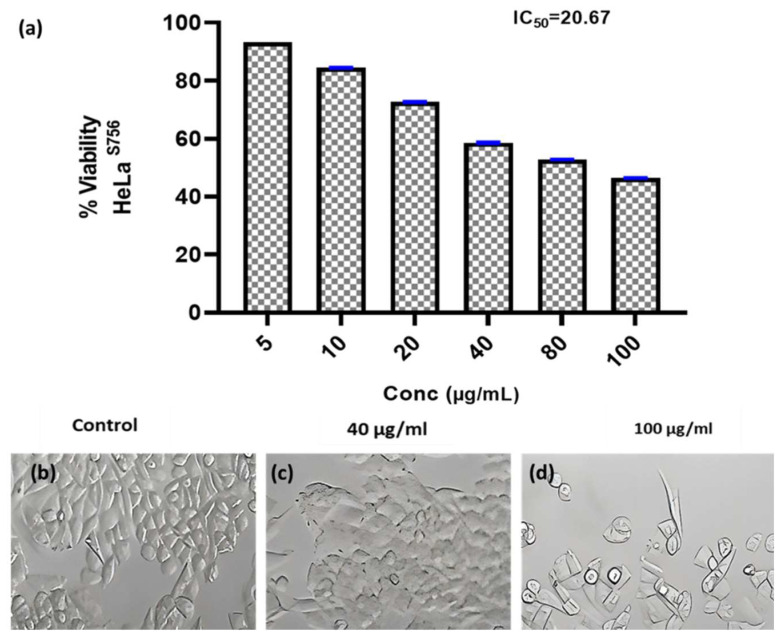
Anticancer activity and IC_50_ of *A. fumigates* WA7S6 against the Hela cell line (**a**). While (**b**) represents the cell line without treatment (control) (**c**,**d**) represent the cell line treated with 40 and 100 µg/mL of fungal extract, respectively. While, The anti-cancer efficacy of the fungal bioactive compounds is clearly evident in both (**c**,**d**).

**Figure 4:**
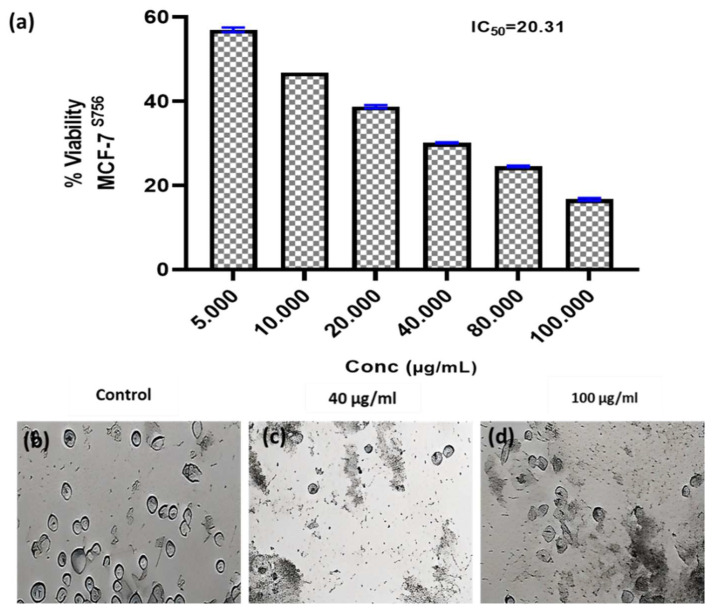
Anticancer activity and IC_50_ of *A. fumigates* WA7S6 against the MCF-7cell line (**a**). While (**b**) represents the cell line without treatment (control), (**c**,**d**) represent the cell line treated with 40 and 100 µg/mL of fungal extract, respectively. While, The anti-cancer efficacy of the fungal bioactive compounds is clearly evident in both (**c**,**d**).

**Figure 5:**
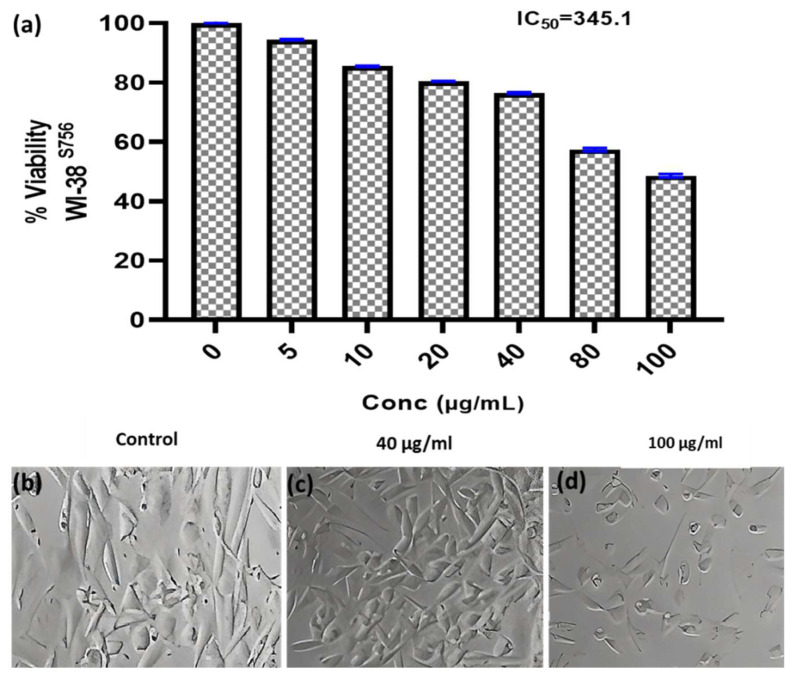
Anticancer activity and IC_50_ of *A. fumigates* WA7S6 against the WI-38 cell line (**a**). While (**b**) represents the cell line without treatment (control), (**c**,**d**) represent the cell line treated with 40 and 100 µg/mL of fungal extract, respectively. The considerably high IC_50_ on the normal cell line suggests the potential utilization of our fungal bioactive compounds, demonstrating a notably favorable safety profile on normal cells.

Researchers have focused a lot of effort on analyzing natural compounds, particularly those derived from fungi, in the search for novel anticancer medications. In this study, the anticancer activity of the fungal crude extract WA7S6 against two well-known cancer cell lines, Hela (cervical cancer) and MCF-7 (breast cancer), was evaluated using a normal cell line, lung fibroblasts (WI-38) (Figure 3, Figure 4 and Figure 5). The primary goal was to determine the (IC_50_), an important metric for evaluating the extract’s ability to stop cell division. The obtained results provided intriguing new details regarding WA7S6’s cytotoxic effects. The IC_50_ values for cancer cell lines were significantly lower than those for normal cell lines. Hela cells had an IC_50_ of 20.67 g/mL, while MCF-7 cells had an IC_50_ of 20.31 g/mL (Figure 4). In contrast, the IC_50_ for WI-38 cells was much higher at 345.1 g/mL. Understanding the anticancer drug WA7S6’s selectivity and possible therapeutic uses depends on these discoveries.

#### 3.6.3. Molecular Docking Study

**Figure 6:**
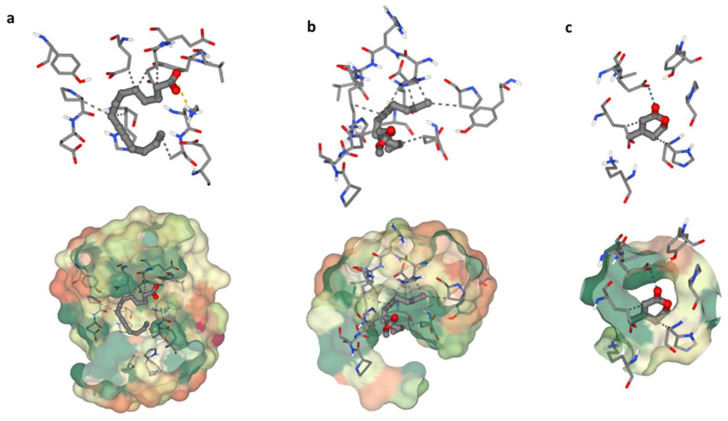
Binding residues of the identified compounds with heme oxygenase (HO-1). The binding residues of the identified compounds with heme oxygenase (HO-1) are demonstrated as follows: (**a**) illustrating the binding interaction of C1 with HO-1, (**b**) showcasing the binding interaction of C2 with HO-1, and (**c**) presenting the binding interaction of C3 with HO-1.

The heme oxygenase, or HO-1 (essential enzyme for oxidative stress) was used in a molecular docking investigation. All discovered compounds were molecular-docked to HO-1 to determine how these compounds interacted with the active site of HO-1 and link the isolated chemicals with the observed activity. Interactions between 9-Tetradecynoic acid and the crystal structure of heme oxygenase (HO-1) have been reported (Figure 6a). The oxygen atom of 9-Tetradecynoic acid was linked to Lys86 via an ionic interaction. A hydrophobic contact was also discovered between C7 and Glu62, C1 and Glu62, C6 and Pro80, C6 and Hsd84, and C16 and Ala87. These associations have a binding energy of −4.7, whereas lower negative values suggest more beneficial and stable binding interactions. Molecular docking research of 11-Hexadecynoic acid, methyl ester, and the heme oxygenase crystal (HO-1) revealed several interactions (Figure 6b). Hydrophobic interactions were found between C11 and Glu62, C14 and Glu62, C1 and Glu66, C16 and Pro80, C16 and Hsd84, and C11 and Lys87 in particular. The resulting binding energy was −4.2 kcal/mol, indicating a positive and sustained interaction. Finally, molecular docking research identified several critical interactions between heme oxygenase crystal structure and dehydromevalonic lactone (Figure 6c). On the other hand, it was discovered that C4 and Glu62 share a hydrophobic relationship, and C1 and Hsd84 have another. A hydrogen bond was also formed between Glu66 and the oxygen atom of dehydromevalonic lactone. As a result of these interactions, the two molecules join together, and the binding energy of −3.9 indicates a durable and beneficial binding arrangement.

## 4. Discussion

Marine fungi have emerged as a significant and largely untapped resource for drug discovery, with a variety of medicinal properties, most notably anticancer and antioxidant activity [28]. Bioactive natural products derived from marine fungi frequently have substantial anti-cancer properties, offering novel treatment options for treating a variety of cancers. The marine endophytic *Aspergillus fumigatus* WA7S6 was isolated from a Hurghada sea sponge and chosen based on antimicrobial screening. The fungus was identified via genotypic analysis, and the resulting gene was placed in Gen-Bank as OR415310.1.

The biological activity of bioactive natural compounds generated from fungi against various cancer cell lines and harmful microorganisms has been the subject of substantial research in recent times. A fungal extract with antibacterial and anticancer properties could work in multiple ways. Apoptosis induction, cell cycle arrest, angiogenesis suppression, and DNA damage in cancer cells are examples of anticancer activity. However, its antibacterial effect is thought to be brought about via inhibition of enzymes that fight bacteria, disruption of cell membranes, suppression of protein synthesis, and disruption of DNA/RNA activities. These advantages are probably caused by the fungus’s bioactive secondary metabolites. However, more research and validation through academic studies are needed for some substances and their particular mechanisms [29].

Several reports have explored the antimicrobial and anticancer properties of fungal bioactive natural products. For instance, the study by Wu et al. [30] highlighted the anticancer and the apoptotic effects of a group of fungal extracts of some different fungal species including *Fomitopsis officinalis*, *Ganoderma sinense, Polyporus melanopus*, and *Taiwanofungus camphorates*, *Fomitopsis pinicola* (*F. pinicola*) toward different cancer cell lines, suggesting a promising source for anticancer and antimicrobial drug development. 

*Aspergillus fumigatus* WA7S6 displayed a pronounced anticancer activity against many cell lines, including hela (cervical cancer) and MCF-7 (breast cancer), while having little cytotoxic activity against the normal cell line, WI-38 (lung fibroblasts). The importance of these findings is that the bioactive component of *Aspergillus fumigatus* WA7S6 has selective toxicity to cancer cells over normal cells. A lower IC_50_ value indicates a stronger inhibitory effect on cell growth. As a result, the extract shows potential for selectively targeting cancer cells while preserving healthy cells, a highly desirable attribute in anticancer therapies to avoid severe side effects. Comparing the IC_50_ values from this investigation to those of similar chemicals or plant extracts with known anticancer characteristics can provide useful insights into the relative efficacy and uniqueness of *Aspergillus fumigatus* WA7S6 as an anticancer agent.

Conversely, the *Aspergillus fumigatus* WA7S6 crude extract showed clear signs of antioxidant activity, with an IC_50_ value of 21.35 µg/mL. Discovering bioactive compounds with antioxidant activity is essential because they minimize the harmful effects of oxidative stress on human health. An imbalance between the body’s capacity to eliminate damaging reactive oxygen species (ROS) with antioxidants and the creation of ROS itself can lead to oxidative stress. A bad diet, smoking, environmental pollutants, and even the body’s regular metabolic activities can all contribute to this imbalance. ROS scavengers such as the antioxidant bioactive substances significantly lessen the damaging effects of ROS on tissues and cells. 

Heme oxygenase, or HO-1, is a crucial enzyme that was used in additional research using computational molecular docking to corroborate the in vitro findings. By cleansing phase II molecules in endothelial cells, HO-1 plays a crucial part in oxidative stress and has been related to possible advantages in heart disease. Furthermore, it is essential in preventing apoptosis, indicating possible cytoprotective benefits [31]. To determine how the discovered compounds interact with the enzyme’s active site and establish a connection between the isolated compounds and the reported activities, a molecular docking experiment was conducted on HO-1 (PDB code: 1N3U).

## 5. Conclusions

In conclusion, the marine fungus *Aspergillus fumigatus* WA7S6, discovered from a sea sponge, appears to have medicinal potentials in a range of areas. It possesses substantial antibacterial and antioxidant capabilities, as well as the potential to act as an anticancer agent. Key molecules with antioxidant activity were found as 9-Tetradecynoic acid, 11-Hexadecynoic acid, methyl ester, and dehydromevalonic lactone. These findings emphasize the varied therapeutic potential of *Aspergillus fumigatus* WA7S6 and its uses in antibacterial, antioxidant, and anticancer drug development.

## Figures and Tables

**Table 1:** Harghada sample data.

Locations	City	Sample Type	Depth (Meter)	Code
Site 1	Hurghada	Seagrass	7.00	SG1
Site 2		Sponge	5.50	SP1
		Sponge	5.50	SP2
		Sponge	5.50	SP3

**Table 2:** Isolation of endophytic fungi from collected marine sea grass and sponges.

Sample Code	Fungus Code	Morphology of Fungus Colony	Crude Extract in (mg)
Seagrass (SG1)	SG1M4	White colony	1.25
	SG1M5	Brown yellowish	1.12
	SG1M6	Black	1.31
	SG1M4	Green	0.98
Sponge (SP1)	SP17S2	White-grey	1.5
	SP17S3	Yellowish-brown	1.23
	SP17S4	Orange	0.69
Sponge (SP2)	WA7S6	Blue-green	1.26
	WA7S7	Black	1.13
Sponge (SP3)	SP27S10	Yellow	0.75
	SP27S11	White	1.62
	SP27S12	Brown	1.5
	SP27S13	Black-brown	1.29

**Table 3:** Antimicrobial inhibitory effect of isolated fungal crude extracts.

Samples Code	Inhibition Ratio (%)				
	*Staphylococcus aureus*	*Salmonella typhi*	*Pseudomonas aeruginosa*	*Candida albicans*	*Aspergillus niger*
SG1M4	50.30 ± 0.23	0	0	0	11.00 ± 0.23
SG1M5	21.02 ± 0.12	0	35.00 ± 0.17	11.22 ± 0.12	9.00 ± 0.15
SG1M6	14.00 ± 0.15	0	16.26 ± 0.23	13.00 ± 0.35	25.00 ± 0.32
SG1M7	0	0	0	0	0
SP17S2	13.37 ± 0.25	0	30.20 ± 0.12	50.35 ± 0.23	23.00 ± 0.12
SP17S3	0	0	0	0	0
SP17S4	0	0	0	0	0
WA7S6	75.55 ± 0.21	0	84.31 ± 0.35	45.25 ± 1.26	65.42 ± 0.24
WA7S7	0	0	0	0	0
SP27S10	0	0	0	0	0
SP27S11	0	0	0	23.20 ± 0.36	32.02 ± 0.13
SP27S12	42.33 ± 0.50	0	0	15.35 ± 0.23	12.23 ± 0.27
SP27S13	35.17 ± 0.21	0	29.23 ± 0.18	36.53 ± 0.12	23.32 ± 0.12
Cip	99.25 ± 0.15	-	98.21 ± 0.26	-	-
Nys	-	-	-	98.23 ± 0.16	98.45 ± 0.26

Cip: Ciprofloxacin (10 µg/mL), Nys: Nystatin (5 µg/mL), -: not detected.

## Data Availability

The datasets used and/or analyzed during the current study are available from the corresponding author upon reasonable request.

## Supplementary Materials

The following supporting information can be downloaded at: https://www.mdpi.com/article/10.3390/microorganisms12010127/s1.
