# Supplementary material for: Anti-Cancer and Anti-Oxidant Bioactive Metabolites from Aspergillus fumigatus WA7S6 Isolated from Marine Sources: In Vitro and In Silico Studies
Source: Microorganisms. 2024 Jan 8;12(1):127. doi: 10.3390/microorganisms12010127 (PMC10819583; doi:10.3390/microorganisms12010127)
Supplement: Supplementary file 1 [file microorganisms-12-00127-s001.zip › microorganisms-2753835-supplementary.pdf]

Anti-Cancer and Anti-Oxidant Bioactive Metabolites from *Aspergillus fumigatus* WA7S6 Isolated from Marine Sources: In Vitro and In Silico Studies

Mervat G. Hassan [1], Waleed A. Elmezain [1], Dina M. Baraka [1], Sabah A. AboElmaaty [1], Ahmed Elhassanein [2,*], Riyad Mohammed Ibrahim [2] and Ahmed A. Hamed [3,*]

1 Botany and Microbiology Department, Faculty of Science, Benha University, Benha 33516, Egypt; mervat.hassan@fsc.bu.edu.eg (M.G.H.); waliedahmed89@yahoo.com (W.A.E.); dina.barakah@fsc.bu.edu.eg (D.M.B.); sabah.alsayed@fsc.bu.edu.eg (S.A.A.)
2 Department of Mathematics, College of Science, University of Bisha, P.O. Box 551, Bisha 61922, Saudi Arabia; rmebrahim@ub.edu.sa
3 Microbial Chemistry Department, National Research Centre, El-Buhouth St. 33, Cairo 12622, Egypt
* Correspondence: el_hassanein@yahoo.com (A.E.); ahmedshalbio@gmail.com (A.A.H.)

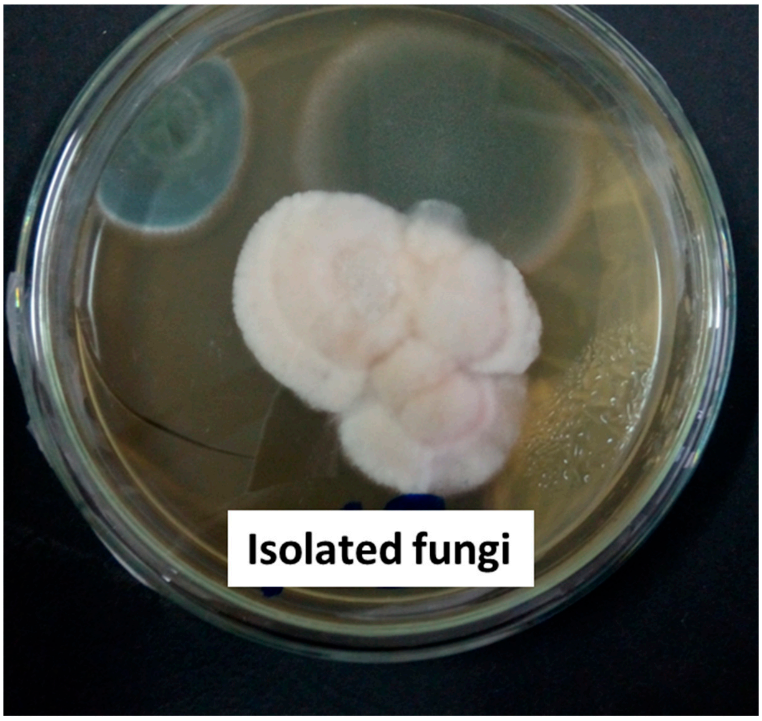

Supplementary S1. Morphology of some isolated fungi

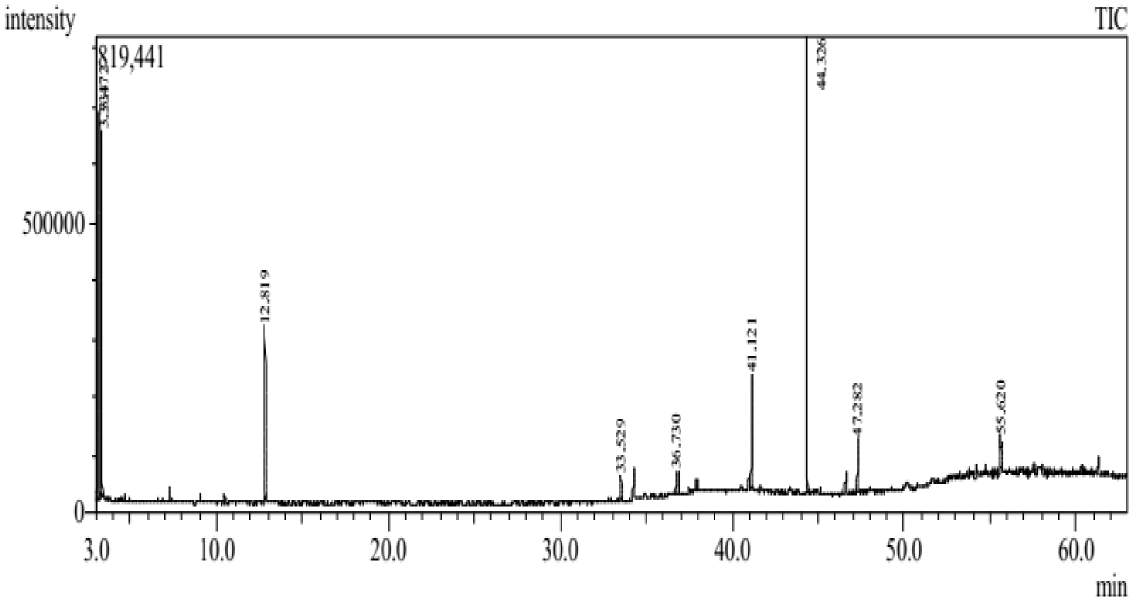

Peak Report TIC

Peak#	R.Time	Area	Area%	Name	Base m/z
1	3.172	1266869	18.68	2,3-Butanediol, [S-(R*,R*)]-	45.10
2	3.334	1137950	16.78	2,3-Butanediol, [S-(R*,R*)]-	45.10
3	12.819	854510	12.60	Dehydromevalonic lactone	82.05
4	33.529	117759	1.74	Hexadecanoic acid, methyl ester	87.10
5	36.730	106851	1.58	9,12-Octadecadien-1-ol, (Z,Z)-	95.15
6	41.121	587929	8.67	13-Docosenamide, (Z)-	59.05
7	44.326	2233614	32.94	Bis(2-ethylhexyl) phthalate	149.00
8	47.282	286447	4.22		307.10
9	55.620	189480	2.79	Card-20(22)-enolide, 3,5,14,19-tetrahydroxy-, (3.beta.,5.beta.)-	95.10

Supplementary S2. GC mass chromatogram of *Aspergillus fumigatus sp*. WA7S6
